# Integrated transcriptomic and metabolomic analysis reveals the effects of EMMPRIN on nucleotide metabolism and 1C metabolism in AS mouse BMDMs

**DOI:** 10.3389/fmolb.2024.1460186

**Published:** 2025-03-07

**Authors:** Yun Zhang, Diyuan Zhang, Zulong Xie, Tianli Xia, Lili Zou, Tao Wang, Li Zhong, Zhuo Zeng, Lingying Wang, Guozhu Chen, Xing Liang

**Affiliations:** ^1^ First Clinical College, Chongqing Medical University, Chongqing, China; ^2^ Second Clinical College, Chongqing Medical University, Chongqing, China; ^3^ Department of Cardiology, The Second Affiliated Hospital of Chongqing Medical University, Chongqing, China

**Keywords:** EMMPRIN, atherosclerosis, metabolomic, transcriptomic, BMDMs, nucleotide metabolism, one-carbon metabolism

## Abstract

**Background:**

Extracellular matrix metalloproteinase inducer (EMMPRIN) has been considered as a key promoting factor in atherosclerosis (AS). Some studies have shown that regulating EMMPRIN expression in bone marrow-derived macrophages (BMDMs) of ApoE−/− mice can affect plaque stability, but the mechanism was not clear.

**Methods:**

AS model mice were built from high-fat-feeding ApoE −/− mice, and were divided into siE group and CON group. The BMDMs and aortas from AS mice were harvested following *in vivo* treatment with either EMMPRIN short interfering (si)RNA (siEMMPRIN) or negative control siRNA. Transcriptomic and metabolomic profiles were analyzed using RNA-sequencing and Liquid chromatography-tandem mass spectrometry (LC-MS/MS), respectively. The efficacy of siEMMPRIN was assessed through real-time quantitative polymerase chain reaction (RT-qPCR) and Western blotting (WB). Immunofluorescence staining was employed to measure EMMPRIN expression within aortic atherosclerotic plaques. Cell proliferation was monitored using the Cell Counting Kit-8 (CCK8), while flow cytometry was utilized to analyze the cell cycle. Additionally, seahorse analysis and oil red O staining were conducted to verify glucose and lipid metabolism, respectively.

**Results:**

A total of 3,282 differentially expressed metabolites (DEMs) and 16,138 differentially expressed genes (DEGs) were identified between the CON group and siE group. The nucleotide metabolism and one-carbon (1C) metabolism were identified as major altered pathways at both the transcriptional and metabolic levels. Metabolomic results identified increased levels of glycine, serine, betaine and S-adenosyl-L-methionine (SAM) to S-adenosyl-L-homocysteine (SAH) ratio and decreased levels of dimethylglycine (DMG) and SAH in 1C metabolism, accompanied by the accumulation of nucleotides, nucleosides, and bases in nucleotide metabolism. Transcriptomics results shown that Dnmt, Mthfd2 and Dhfr were downregulated, while Mthfr were upregulated in 1C metabolism. And numerous genes involved in *de novo* nucleotide synthesis, pentose phosphate pathway (PPP) and dNTP production were significantly inhibited, which may be associated with decreased BMDMs proliferation and cell cycle arrest in the G0/G1 phase in siE group. Multi-omics results also showed changes in glucose and lipid metabolism. Seahorse assay confirmed reduced glycolysis and oxidative phosphorylation (OXPHOS) levels and the Oil Red O staining confirmed the decrease of lipid droplets in siE group.

**Conclusion:**

The integrated metabolomic and transcriptomic analysis suggested that nucleotide metabolism and 1C metabolism may be major metabolic pathways affected by siEMMPRIN in AS mouse BMDMs. Our study contributes to a better understanding of the role of EMMPRIN in AS development.

## 1 Introduction

Atherosclerosis (AS) is a chronic inflammatory disease characterized by plaque formation in the arterial walls mediated by lipid accumulation (Björkegren and Lusis, 2022). Macrophages play a crucial role in the pathogenesis of AS by participating in both the inflammatory response and foam cell formation (Zhang et al., 2020). Bone marrow-derived monocytes are the main precursors of macrophages in atherosclerotic plaque, which are recruited to the subendothelium from blood and differentiate into macrophages (Zhang et al., 2022). Before reaching the artery wall, monocytes are exposed to a variety of pathological stimuli within the systemic milieu during AS. This exposure leads to changes in cellular metabolism and function, which further affects the occurrence and development of AS (Koelwyn et al., 2018). Therefore, the changes in monocytes/macrophages metabolism may have a crucial impact on the pathological progression of AS.

Extracellular matrix metalloproteinase inducer (EMMPRIN), also known as Basigin or CD147, is a highly glycosylated transmembrane glycoprotein of about 50–60 kDa, belonging to the immunoglobulin superfamily (Siracusano et al., 2020). EMMPRIN is abundantly expressed on the surface of monocytes/macrophages and is involved in AS and atherothrombosis (Wang et al., 2015). Our previous studies demonstrated that EMMPRIN increases AS plaque vulnerability by regulating macrophage autophagy, affecting cell accumulation, the release of matrix metalloproteins (MMPs) and pro-inflammatory factors; maintaining low levels of EMMPRIN expression exerted a protective effect against AS (Liang et al., 2018; Liu et al., 2013). In addition, Lv et al. (2024) and Lv et al. (2021) found that EMMPRIN plays a pro-atherogenic role by facilitating foam cell formation, driving a pro-inflammatory phenotype in macrophages and impairing macrophage efferocytosis, thereby increasing the vulnerability of AS plaque. These findings suggest that EMMPRIN plays a central role in promoting the formation of AS vulnerable plaque. However, the effects of EMMPRIN on monocytes and macrophages metabolism, and hence on AS development, remain unclear.

Several studies have reported that EMMPRIN is involved in the metabolic reprogramming of various tumor cells. Dong et al. (2022) found that EMMPRIN mediated the reprogramming of glycolipid metabolism in colorectal cancer. Li et al. (2020) demonstrated that EMMPRIN promotes glycolytic metabolism in hepatocellular carcinoma (HCC) through the PI3K/Akt/mTOR signaling pathway, as well as its association with immunosuppression in HCC. Additionally, EMMPRIN also plays a significant role in the reprogramming of fatty acid metabolism in HCC, contributing to their proliferation and metastasis (Li et al., 2015). Moreover, except in tumor cells, Kaushik et al. (2019) observed a decrease in glycolysis and proinflammatory functions in macrophages following EMMPRIN knockdown. Our previous research revealed that EMMPRIN can affect the activation of the PI3K/Akt pathway, which is related to metabolism in RAW264.7 cells and plaque of ApoE−/− mice (Liang et al., 2018). These results suggest the potential role of EMMPRIN in AS by regulating macrophage metabolism and function. Nevertheless, the precise metabolic implications of EMMPRIN on bone marrow-derived macrophages (BMDMs) in AS remain unclear.

This study utilized integrated transcriptomic and metabolomic analyses to investigate the effects of EMMPRIN siRNA on the function and metabolite profiles of BMDMs of AS mice. Our findings offer a comprehensive exploration of the impact of EMMPRIN siRNA on metabolic profiles and cellular functions in BMDMs of AS mice, enhancing our understanding of the role of EMMPRIN in the development of AS vulnerable plaques.

## 2 Materials and methods

### 2.1 Reagents

Recombinant mouse macrophage colony-stimulating factor (M-CSF) were purchased from PeproTech (Rocky Hill, NJ, United States). Dulbecco’s modified Eagle’s medium (DMEM), foetal bovine serum, and penicillin-streptomycinliquid were obtained from Thermo Fisher Scientific Inc. (Shanghai, China). A shortinterfering (si)RNA specific to EMMPRIN and negative control siRNA were constructed by Beijing Tsingke Biotech Co., Ltd. (Beijing, China). Monoclonal antibody targeting EMMRPIN (sc53064) were purchased from Santa Cruz Biotechnology, Inc. Monoclonal antibody targeting CD68 (ab283654) and Goat Anti-Rabbit IgG H&L (HRP) (ab6721) were purchased from Abcam (Cambridge, United Kingdom). RIPA lysis buffer (P0013B), BCA kit (P0012S), SDS-PAGE Electrophoresis Buffer (P0014), Soaking and Activation Buffer for PVDF Membrane (P0021S), polyvinylidene difluoride (PVDF) membranes (FFP80), Blocking Buffer for Western blot (P0023B), Antifade Mounting Medium with DAPI (P0131), Immunol Staining Blocking Buffer (P0102), Enhanced Immunostaining Permeabilization Buffer (P0097), TBS with Tween-20 (ST673), anti-GAPDH (AF1186), Cell Counting Kit-8 (CCK8) (C0041) and Oil Red O Staining kit (C0157M) were obtained from Beyotime Institute of Biotechnology, Inc (Shanghai, China). ECL Western blotting Detection Kit (SW2030) were obtained from Solarbio (Beijing, China). Cell Cycle Assay Kit (E-CK-A351) were obtained from Elabscience Biotechnology, Inc. (Wuhan, China). TRIzol reagent, PrimeScript II First-Strand cDNA Synthesis Kit were obtained from TaKaRa Bio, Inc. (Otsu, Japan). XF DMEM base medium (Cat No. 103575-100), XF Glycolysis Rate Assay Kit (Cat No. 103344-100) and XF Cell Mito Stress Assay Kit (Cat No. 103015-100) were purchased from Agilent Technologies, Inc. (California, United States).

### 2.2 Animals

The Experimental Animal Ethics Committee (ChongQing Medical University) approved all animal procedures, which were performed in accordance with the Guide for the Care and Use of Laboratory Animals published by the U.S. National Institute of Health (NIH Publication No. 85-23, revised 1996).

A total of 18 male ApoE−/− mice (8 weeks old, 20–22 g, C57BL/6J genetic background, Beijing University, China) were maintained under specific pathogen-free (SPF) conditions at 22°C under a 12/12-h light/dark cycle and received drinking water *ad libitum*.

For the preparation of the study, 6 mice were randomly divided into 2 groups as follows: CON (High fat diet + negative control siRNA, n = 3), siE (High fat diet + EMMPRIN siRNA, n = 3) and fed with a high fat diet (Western diet that contained 15% fat from lard and was supplemented with 1.25% (w/w) cholesterol) for 12 weeks. Starting from week 13, mice were intervened by tail vein injection with negative control siRNA or EMMPRIN small interfering RNA (siEMMPRIN) (4OD, twice per week, 4 weeks) into the mice (same diet was continued from 13 to 16 weeks of feeding). At the end of the treatment, 6 animals were anesthetized, and the femurs, tibias and thoracic aortas were harvested. They were used for transcriptomic analysis, metabonomic analysis, Western blotting (WB), real-time quantitative polymerase chain reaction (RT-qPCR) and immunofluorescence staining. For next stage experiment, 12 mice were randomly divided into 2 groups as follows: CON (High fat diet + negative control siRNA, n = 6), siE (High fat diet + EMMPRIN siRNA, n = 6) and fed with a high fat diet (Western diet that contained 15% fat from lard and was supplemented with 1.25% (w/w) cholesterol) for 12 weeks. Starting from week 13, mice were intervened by tail vein injection with negative control siRNA or EMMPRIN small interfering RNA (4OD, twice per week, 4 weeks) into the mice (same diet was continued from 13 to 16 weeks of feeding). At the end of the treatment, 12 animals were anesthetized, and the femurs, tibias and thoracic aortas were harvested. They were used to detect cell proliferation, cell cycle, Oil red O staining and seahorse analysis.

### 2.3 Bone marrow-derived macrophages culture

Bone marrow cell suspensions were isolated from each ApoE−/− mouse by flushing the femurs and tibias with PBS. Cell suspensions were passed through a 70 μm cell strainer, collected by centrifugation at 300 g for 10 min, and resuspended in DMEM containing 10% FBS, 100 U/mL penicillin, 100 mg/mL streptomycin and 50 ng/ml M-CSF. After 24 h, the adherent cells were removed and the suspended cells were collected for further culture. Cells continue to be cultured at 37°C with 5% CO_2_ for 6 days. Expression of F4/80 was examined by flow cytometry to determine the purity of BMDMs.

### 2.4 Western blotting (WB)

The protein expression of EMMPRIN in aortic plaque tissue and BMDMs of AS model mice was measured by WB. Total proteins from the aortic tissue and BMDMs samples were measured using a BCA protein assay kit. Proteins were separated using SDS gel electrophoresis and transferred to PVDF membranes. The membranes were blocked by Blocking Buffer for Western blot for 2 h and incubated overnight at 4°C with the following primary antibodies: anti-EMMRPIN (1:1000), anti-GAPDH (1:2000). After washing with TBST, the membranes were incubated with the secondary antibody, goat anti-rabbit IgG H&L (HRP) (1:10000). The membranes were developed with ECL Western blotting Detection Kit, and the band intensity was determined by ImageJ software (National Institutes of Health, United States).

### 2.5 Quantitative real-time PCR (qRT-PCR)

The gene expressions of EMMPRIN were detected by RT-qPCR. Total RNA was extracted from BMDMs using TRIzol reagent. RNA was reverse-transcribed into cDNA using a PrimeScript II First-Strand cDNA Synthesis Kit. RT-qPCR was carried out using CFX96 Real-Time PCR Detection System (Bio-rad, Minneapolis, MN, United States). The primer of EMMPRIN is 5′GCAGAGGACACAGGCACTTAC 3′and 5′ACAGGCTCAGGAAGGATG 3′. The relative expression of EMMPRIN gene was determined using the Comparative CT (△△Ct) method, and was normalized to the expression of β-actin gene.

### 2.6 Immunofluorescence staining

The frozen sections of the thoracic aortas were washed with PBS and pre-incubated with a permeabilisation/blocking buffer for 30 min at room temperature. Then the sections were incubated with a goat anti-mouse EMMPRIN antibody (1:200) at 4°C overnight. The slides were washed with PBS andincubated with a rabbit anti-CD68 (1:500) at 4°C overnight. The slides were washed three times with PBS for 10 min each time and incubated with the corresponding fluorescent-labelled secondary antibodies (1:5000) at room temperature for 2 h in complete darkness. The cell nuclei were counterstained via incubation with 4′-6-diamid ino-2-phenylindole (DAPI, 1 mg/mL). Finally, the slides were analysed under a confocal microscope (ZEISS, Germany). The images were digitally analysed using the ZEN microsystem software (Carl Zeiss Microscopy GmbH).

### 2.7 Seahorse analysis

Real-time changes in extracellular acidification rate (ECAR) and oxygen consumption rate (OCR) of BMDMs were analyzed with an XF-96 Extracellular Flux Analyzer. Briefly, BMDMs were seeded into XF96 Cell Culture Microplates at a density of 1 × 105 cells per well and cultured overnight. The culture medium was switched to XF DMEM base medium supplemented. The ECAR and OCR were measured using an XFe96 analyzer after sequential injection of the compounds of the XF Glycolysis Rate Assay Kit or the XF Cell Mito Stress Assay Kit. The ECAR and OCR were automatically calculated by Seahorse XFe96 software.

### 2.8 Oil red O staining

BMDMs were fixed with 4% paraformaldehyde for 10 min at room temperature, and were stained using Oil Red O (30 min, 60°C). After staining, red fat droplets in macrophages were observed by phase contrast microscopy.

### 2.9 Cell proliferation and cell cycle

BMDMs were collected from the cultures and re-plated into 96-well plates (1 × 102 cells/mL). Cell proliferation was measured using a Cell Counting Kit-8 assay according to the manufacturer’s protocol. The percentage of cells in each phase of the cell cycle were determined using cell cycle assay kit by flow cytometry.

### 2.10 Transcriptome analysis (RNA-seq analysis)

Six RNA samples of CON and siE group mouse BMDMs were sent for sequencing in BioTree (BioTree, Shanghai, China). Total RNA was extracted using TRIzol reagent (thermofisher, 15596018) kit according to the manufacturer’s protocol. The total RNA quantity and purity were analysis of Bioanalyzer 2100 and RNA 6000 Nano LabChip Kit (Agilent, CA, United States). Subsequently, the mRNA was purified from total RNA (5 μg) using Dynabeads Oligo (dT) (Thermo Fisher, CA, United States) with two rounds of purification. Following purification, the mRNA was fragmented into short fragments using divalent cations. Then, the fragments were reverse-transcribed to create the cDNA using random primers. The cDNA underwent a series of synthesis, modification, conversion, and PCR amplification processes to form libraries with fragment average sizes of 300 bp ± 50 bp (strand-specific libraries). At last, we performed the 2 × 150 bp paired-end sequencing (PE150) on an Illumina Novaseq™ 6000 following the vendor’s recommended protocol. The gene expression abundance and variations were analyzed by calculating FPKM (fragment per kilobase of transcript per million mapped reads) value by StringTie software. The genes with the parameters of p-value <0.05 and absolute |log2fold change (FC)| ≥1 were considered as differentially expressed genes (DEGs) performed by DESeq2 software. The identified DEGs were then subjected to enrichment analysis of Gene Ontology (GO) functions and Kyoto Encyclopedia of Genes and Genomes (KEGG) pathways using the clusterProfiler package in R, with adjusted p-value <0.05 considered significantly enriched.

### 2.11 Metabonomic analysis

BMDMs from six samples of CON and siE group mouse BMDMs were subjected to untargeted cell metabolomics analysis conducted by BioTree (BioTree, Shanghai, China). The pellets of BMDMs (about 107 cells) were combined with 1,000 μL of extraction solution (MeOH: ACN: H2O, 2:2:1 (v/v)) containing deuterated internal standards. The mixture was vortexed for 30s and incubated in liquid nitrogen for 1 min. The samples were thawed at room temperature and vortexed for 30s, with this freeze-thaw cycle being repeated three times. Subsequently, the samples were sonicated for 10 min in a 4°C water bath and incubated at −40°C for 1 h to precipitate proteins. The samples were centrifuged at 12,000 rpm (RCF = 13,800 (×g), R = 8.6 cm) for 15 min at 4°C. The supernatant was transferred to a fresh glass vial for further analysis. Liquid chromatography-tandem mass spectrometry (LC-MS/MS) analyses of polar metabolites were conducted using an UHPLC system (Vanquish, Thermo Fisher Scientific) coupled to Orbitrap Exploris 120 mass spectrometer (Orbitrap MS, Thermo) and equipped with a Waters ACQUITY UPLC BEH Amide (2.1 mm × 50 mm, 1.7 μm). The mobile phase consisted of 25 mmol/L ammonium acetate and 25 mmol/L ammonia hydroxide in water (pH = 9.75) (A) and acetonitrile (B). The auto-sampler temperature was set to 4°C, with an injection volume of 2 μL. The raw data were converted to mzXML format using ProteoWizard and processed with an in-house program developed using R and based on XCMS for peak detection, extraction, alignment, and integration. The R package and BiotreeDB (V3.0) were utilized for metabolite identification. Multivariate statistical analysis, specifically Orthogonal Partial Least Squares Discriminant Analysis (OPLS-DA), was employed to identify metabolites exhibiting significant differences. The significance threshold was set at VIP >1 and a P-value <0.05. The significantly different metabolites were then subjected to KEGG pathway enrichment analysis.

### 2.12 Integrated network analysis of transcriptomics and metabolomics

KEGG (http://www.genome.jp/kegg/) pathway maps link differentially expressed genes (DEGs) and differentially expressed metabolites (DEMs) in the transcriptome and metabolome, providing a method for integrated transcriptional and metabolic analyses and constructing intergroup linkage networks. All DEGs and DEMs in this study were collated and mapped to the KEGG pathway enrichment database to obtain their common pathway information. The transcript-metabolite regulatory networks were visualized using the R (vision 4.3.2).

### 2.13 Statistical analysis

Statistical analysis was performed using GraphPad Prism version 9.5 (GraphPad, CA). Results were presented as mean ± standard deviation (SD) and compared between groups using Student’s t-test or two-way ANOVA. P-value <0.05 was considered significant and indicated by *P < 0.05, **P < 0.01, and ***P < 0.001.

## 3 Results

### 3.1 The expression of EMMPRIN was significantly reduced in BMDMs by siRNA

**FIGURE 1:**
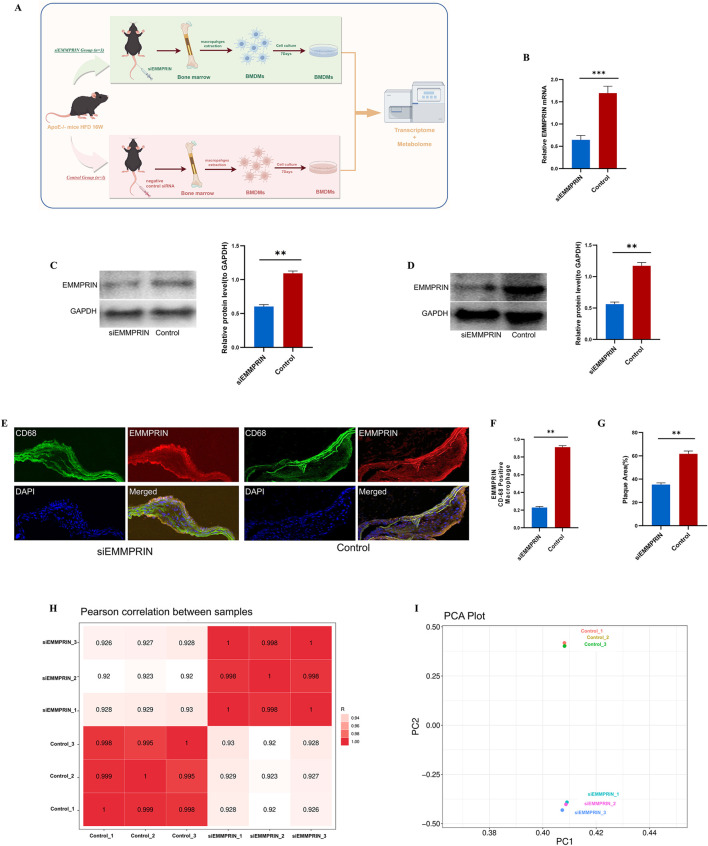
The expression of EMMPRIN was significantly reduced in BMDMs by siRNA. **(A)** Study strategy for transcriptome and metabolome of siE (n = 3) and CON (n = 3). BMDMs were extracted and cultured for 7 days and were processed for RNA-seq analysis and metabolomic analysis **(B)** RT-qPCR analysis of BMDMs induced by siEMMPRIN (n = 3) and controls (n = 3). **(C)** WB for validating the silencing of EMMPRIN in BMDMs (n = 3 for each group). **(D)** WB for validating the silencing of EMMPRIN in atherosclerotic plaque in ApoE−/−mice (n = 3 for each group) **(E)** Immunofluorescence showed the expression of EMMPRIN in CD68-positive macrophage in aortic atherosclerotic plaque (n = 3 for each group). **(F)** The aortic atherosclerotic plaque area showed a significant decrease in siEMMPRIN group (n = 3 for each group). **(G)** Expression of EMMPRIN presented significant decrease (n = 3 for each group). **(H)** Heat map of Pearson correlation analysis between the two groups. **(I)** Diagram of PCA analysis.

The transcriptome and metabolome research strategies employed in this study are illustrated in Figure 1A. The RNA expression of EMMPRIN in BMDMs of AS model mice was detected by RT-qPCR. The results indicated that siEMMPRIN intervention resulted in a significant reduction of EMMPRIN gene expression by over 50% in BMDMs, compared to the CON group (Figure 1B). Furthermore, WB analysis demonstrated that the protein expression of EMMPRIN decreased after siEMMPRIN intervention (Figure 1C). These results suggest that EMMPRIN siRNA administration effectively inhibits EMMPRIN expression in BMDMs of AS model mice.

We also investigated EMMPRIN expression in aortic atherosclerotic plaque by using WB. The expression of EMMPRIN in the siE group was lower than that in the CON group (Figure 1D). Furthermore, EMMPRIN expression in CD68-positive macrophage in aortic atherosclerotic plaque was detected by Immunofluorescence. The expression of EMMPRIN in the plaques of the siE group was significantly lower than that of the CON group, accompanied by a significant reduction in plaques in the siE group compared to the CON group (Figures 1E–G).

### 3.2 Transcriptomic changes in AS mouse BMDMs induced by siEMMPRIN intervention

**FIGURE 2:**
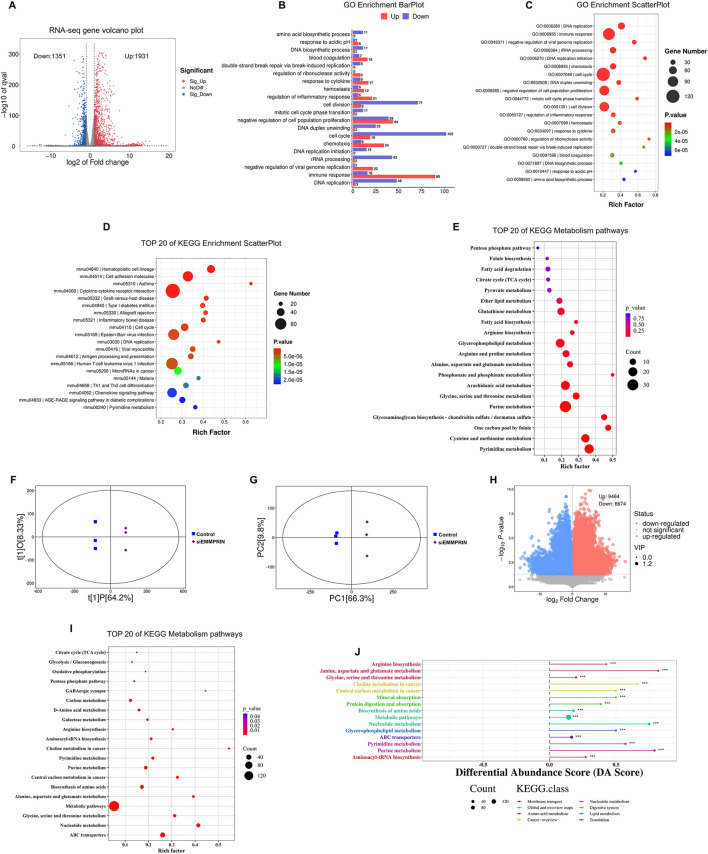
Transcriptome and metabolomics analysis following siEMMPRIN treatment. **(A)** The volcano plot of DEGs (FC > 2 and P < 0.05) between siE and CON gourp. The grey dots represent genes without significant difference between the two groups; the red dots represent significantly upregulated DEGs in siE group and the blue dots indicate significantly downregulated ones. **(B)** Biological processes terms scatter plot from the GO enrichment analysis based on DEGs between the two groups. **(C)** Up- and downregulation histograms of related genes in biological processes from the GO enrichment analysis. **(D)** The top 20 KEGG pathways based on the DEGs. **(E)** The top 20 KEGG metabolic relevant pathways of DEGs. **(F)** OPLS-DA analysis of siE and CON group. **(G)** PCA analysis between the two groups. **(H)** The volcano plot of DEMs (VIP >1 and P < 0.05) between the two groups. **(I)** The top 20 KEGG metabolic pathways of DEMs. **(J)** DA score analysis plot of DEMs.

Transcriptome analysis was performed to explore the gene expression of EMMPRIN in two groups to elucidate the underlying molecular mechanism in BMDMs. The high Pearson correlation coefficients exceeding 0.920 between the siE and the CON groups indicated the reliability of the sequencing results (Figure 1H). Principal component analysis (PCA) was utilized to cluster the samples, revealing a clear separation between the two groups (Figure 1I), siEMMPRIN exerted a significant impact on the gene expression of BMDMs between the two groups. A total of 3,282 DEGs were identified, of which 1,931 were upregulated and 1,351 were downregulated (Figure 2A).

GO and KEGG pathway enrichment analysis was performed to investigate the biological functions of the identified DEGs. A total of 1,466 significant GO terms were found (P. adj< 0.05). The results suggested an obvious enrichment and downregulation of GO terms related to cell proliferation, cell cycle, DNA replication, and cell division in the siE group (Figures 2B, C). These findings suggest that the proliferation of BMDMs in the siE group may be inhibited. In the KEGG pathway enrichment analysis, DEGs were predominantly associated with hematopoietic cell lineage, cell adhesion molecules, and cell cycle among other pathways (Figure 2D). To further elucidate the metabolic alterations, a total of 75 KEGG pathways related to metabolism were selected. In addition, the top 20 metabolic pathways are shown in Figure 2E, including pyrimidine metabolism, one carbon pool by folate, cysteine-methionine metabolism, purine metabolism, and glycine-serine-threonine metabolism.

The transcriptomic results provided initial insights into the changes in biological processes in BMDMs due to decreased EMMPRIN expression. These were primarily associated with inhibition of cell cycle regulation and DNA replication, as well as significant metabolic changes.

### 3.3 Untargeted metabolomic changes in AS mouse BMDMs induced by siEMMPRIN intervention

LC-MS/MS analytical techniques were used to detect changes in off-target metabolites to detect changes in metabolic profiles in AS mouse BMDMs induced by siEMMPRIN. PCA and OPLS-DA were performed and revealed a significant difference in the distribution between the siE and CON group (Figures 2F, G). Furthermore, the metabolomic results indicated significant differences in the composition and quantity of metabolites between the two groups. After relative standard deviation de-noising, a total of 16,138 DEMs were detected, of which 9,464 were upregulated and 6,674 were downregulated (Figure 2H).

To further explore the potential metabolic pathways of BMDMs induced by siEMMPRIN, DEMs were annotated and assigned using the KEGG database, showing a total of 149 enriched KEGG pathways. Among these, ABC transporters, nucleotide metabolism, glycine-serine-threonine metabolism, biosynthesis of amino acid, purine metabolism, pyrimidine metabolism, and carbon metabolism were significantly enriched (Figure 2I). The DA analysis demonstrated 15 positively scored pathways, including glycine-serine-threonine metabolism, central carbon metabolism in cancer, purine metabolism, pyrimidine metabolism, carbon metabolism and ABC transporters (Figure 2J).

### 3.4 Integrated transcriptomics and metabolomics revealed significant changes in nucleotide metabolism, amino acid metabolism, glucose metabolism, and lipid metabolism in AS mouse BMDMs induced by siEMMPRIN intervention

**FIGURE 3:**
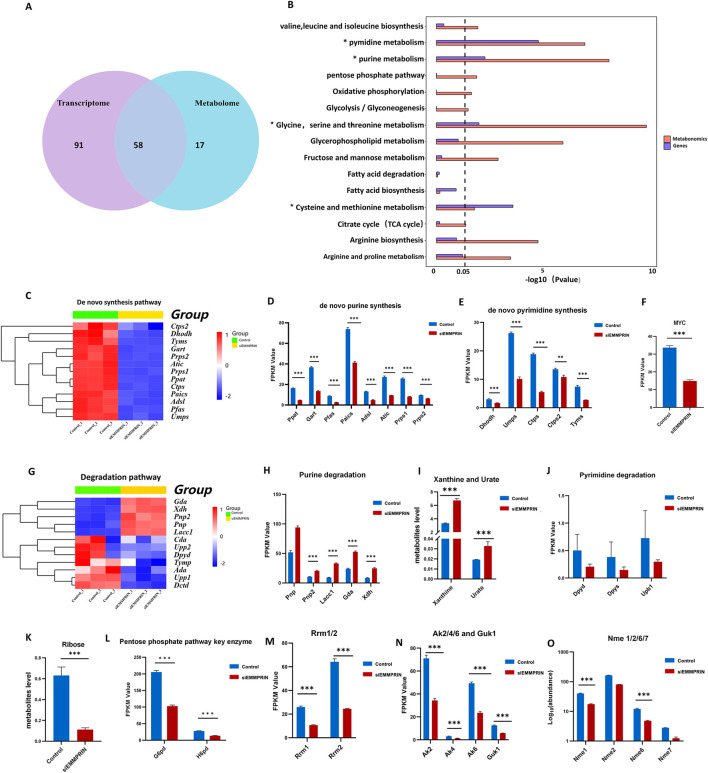
Integrated multi-omics analysis and changes in nucleotide metabolism. **(A)** Diagram of KEGG metabolic pathways enriched in both transcriptomics and metabolomics. **(B)** Common metabolic pathways of transcriptome and metabolome KEGG analysis based on DEGs and DEMs. **(C)** Heat map of nucleotide *de novo* synthesis gene expression. **(D, E)** Diagram of genes related to purine and pyrimidine *de novo* synthesis. P-value was indicated by *P < 0.05, **P < 0.01, and ***P < 0.001. **(F)** Myc expression was downregulated in the transcriptome. **(G)** Heat map of purine and pyrimidine degradation pathway gene expression. **(H)** Diagram of genes related to the purine degradation pathway. **(I)** Xanthine and urate were downregulated in purine degradation metabolites in the siE group. **(J)** Diagram of genes related to the pyrimidine degradation pathway. **(K)** Ribose levels were downregulated in metabolites. **(L)** G6pd and H6pd expression were downregulated. **(M)** Rrm1 and Rrm2 levels were downregulated. **(N)** Ak2, Ak4, Ak6 and Guk1 were downregulated. **(O)** Nme levels were downregulated.

Integrated analysis of KEGG pathways was performed using transcriptomics and metabolomics data, comprehensively elucidating the changes in biological metabolic processes of BMDMs. Transcriptomics and metabolomics revealed that 58 pathways were enriched, of which four were statistically significant, including purine metabolism, pyrimidine metabolism, glycine-serine-threonine metabolism, and cysteine-methionine metabolism (Figures 3A, B). Additionally, changes in glucose metabolism and lipid metabolism were observed in transcriptomics and metabolomics.

#### 3.4.1 Nucleotide metabolism is significantly altered in AS mouse BMDMs induced by siEMMPRIN

Integrative analyses revealed that purine and pyrimidine metabolism were significantly altered in the siE group. In the transcriptome, relevant genes of the purine *de novo* synthesis pathway were significantly downregulated, including Prps1, Prps2, Ppat, Gart, Pfas, Paics, Adsl, and Atic (Figures 3C, D). Moreover, downregulation of *de novo* synthesis-related genes was observed in pyrimidine metabolism, including Dhodh, Umps, Ctps, Ctps2, and Tyms (Figures 3C, E). These results indicated that both the purine and pyrimidine nucleotide *de novo* synthesis pathways were inhibited in the siE group. MYC is an important upstream regulator of purine and pyrimidine *de novo* synthesis, which was also downregulated (Figure 3F). Genes associated with nucleotide salvage synthesis, such as Aprt, Hprt, and Adk, showed no significant changes. The genes related to purine degradation, including Pnp2, Lacc1, Gda, and Xdh, were significantly upregulated (Figures 3G, H). In addition, elevated levels of purine degradation metabolites, such as xanthine and urate, were detected (Figure 3I). However, no obvious changes were found in the genes and metabolites associated with the pyrimidine nucleotide degradation pathway (Figure 3J). Furthermore, ribose is an important raw material for nucleotide production mainly through the pentose phosphate pathway (PPP). The metabolome results suggested a significant decrease in the level of ribose in the siE group (Figure 3K). The transcriptome results showed decreased expression of G6pd and H6pd in the siE group, which are key enzymes of the PPP (Figure 3L).

**FIGURE 4:**
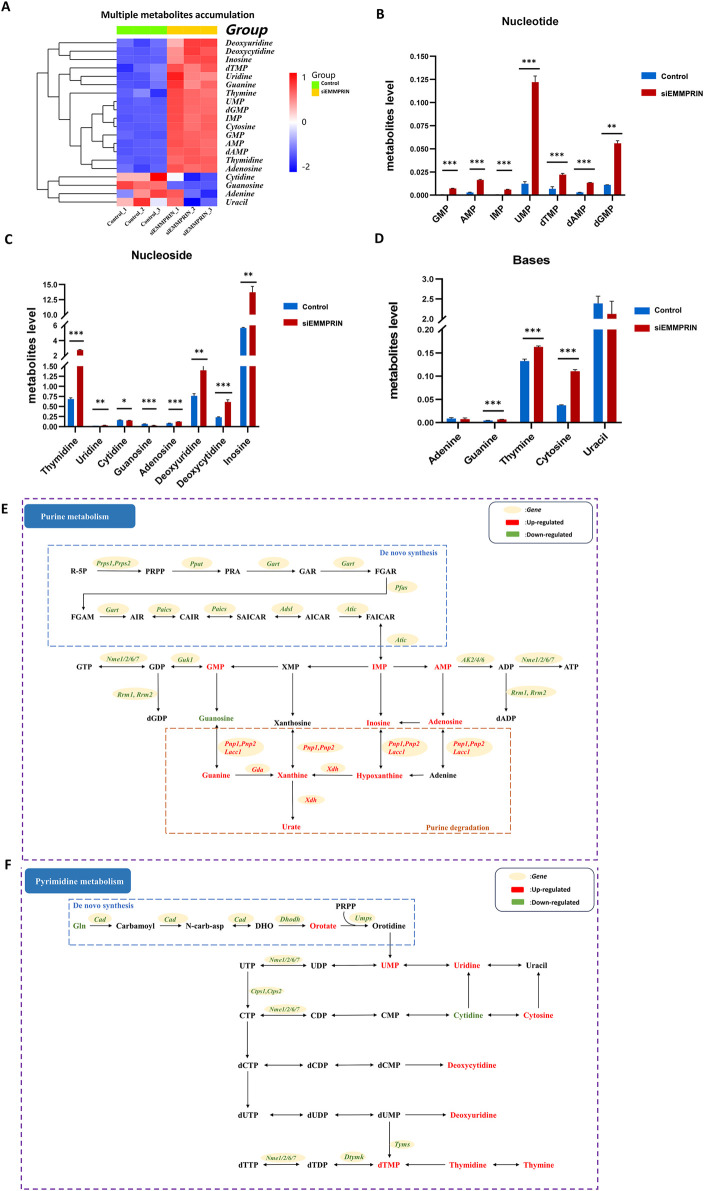
Metabolites changes in nucleoside metabolism. **(A)** Heat map of obvious accumulation of nucleotides, nucleosides, and bases in nucleoside metabolism. **(B–D)** Diagram showing the accumulation of multiple nucleotides, nucleosides, and bases respectively. **(E)** Diagram of purine metabolism. **(F)** Diagram of pyrimidine metabolism. The red color indicates upregulated genes or metabolites in BMDMs induced by siEMMPRIN, while the green one indicates downregulated.

Additionally, the levels of Rrm, encoding ribonucleotide reductase (RNR), were significantly downregulated (Figure 3M). Nucleoside diphosphate kinase (NMPK), such as guanylate kinase (GMK) and adenylate kinase (AMK), is responsible for the conversion of NDP to NTP. The levels of Ak and Guk1, encoding AMK and GMK, respectively, were significantly downregulated (Figure 3N). Moreover, the levels of Nme1 and Nme6 encoding nucleoside diphosphate kinase (NDPK), which is responsible for the conversion of NDP to NTP, were significantly downregulated (Figure 3O). In addition, our results show a significant accumulation of nucleotides, nucleosides, and bases in nucleoside metabolism (Figure 4A). In detail, the GMP, AMP, UMP, IMP, dTMP, dAMP, and dGMP were significantly upregulated (Figure 4B). Meanwhile, partial nucleosides were also upregulated, such as thymidine, uridine, deoxycytidine, deoxyuridine, inosine, and adenosine (Figure 4C). As for the bases, only the thymine, guanine, and cytosine levels were significantly upregulated (Figure 4D).

In summary, nucleotide synthesis-related genes were widely downregulated, and various metabolic intermediates accumulated in large quantities, showing a trend of increased degradation. These results suggested that nucleotide metabolism was significantly altered in the siE group (Figures 4E, F).

#### 3.4.2 One-carbon (1C) metabolism was significantly altered in AS mouse BMDMs treated with siEMMPRIN

**FIGURE 5:**
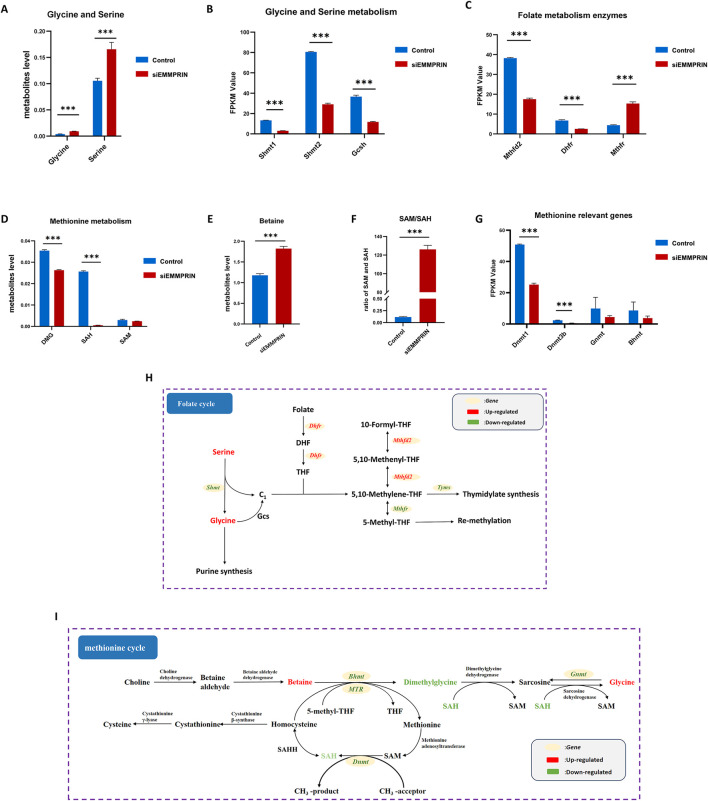
Genes expression and metabolites of one-carbon metabolism. P-value was indicated by *P < 0.05, **P < 0.01, and ***P < 0.001. **(A)** Upregulation of glycine and serine in BMDMs induced by siEMMPRIN. **(B)** Expression of genes related to glycine and serine metabolism. **(C)** Expression of genes related to 1C unit cycle metabolism. **(D, E)** Differential expression of methionine cycle metabolites including DMG, SAH, and Betaine. **(F)** The ratio of SAM/SAH was significantly upregulated. **(G)** Expression of genes related to methionine cycle. **(H, I)** Diagram of folate cycle and methionine cycle. The red and green colors indicate genes or metabolites that were upregulated and downregulated in BMDMs induced by siEMMPRIN, respectively.

Our findings revealed notable changes in the glycine-serine-threonine metabolism, cysteine-methionine metabolism, and folate metabolism, which are key components of the 1C metabolism. The metabolomic results showed increased levels of glycine and serine, and the transcriptomic results suggested decreased levels of Shmt1, Shmt2, and Gcsh gene expression (Figures 5A, B). Glycine cleavage system H protein (GCSH) is a component of the glycine cleavage system (GCS). Serine and glycine contribute 1C units to the folate cycle via serine hydroxymethyltransferase (SHMT) and GCS, respectively. This suggested that inhibition of EMMPRIN gene may reduce the production of 1C units in BMDMs. Moreover, the levels of genes associated with the folate metabolizing enzymes Mthfd2 and Dhfr were downregulated, while Mthfr was increased (Figure 5C). This suggested that inhibition of EMMPRIN gene may lead to compromised folate cycling in BMDMs. In relation to the methionine cycle, metabolomic results identified increased levels of betaine and decreased levels of dimethylglycine (DMG) and S-adenosyl-L-homocysteine (SAH) (Figures 5D, E). Moreover, S-adenosyl-L-methionine (SAM) exhibited a trend towards downregulation (Figure 5D). Furthermore, the SAM to SAH ratio, which is strongly correlated with the methylation response, was significantly upregulated in our results (Figure 5F). The transcriptomics results revealed that the levels of genes for enzymes related to the methionine cycle were affected, including Dnmt, Bhmt, and Gnmt (Figure 5G). Collectively, siEMMPRIN may significantly affect folate cycle and methionine metabolism responses in BMDMs (Figures 5H, I).

#### 3.4.3 Glucose metabolism and lipid metabolism changed in AS mouse BMDMs induced by siEMMPRIN

**FIGURE 6:**
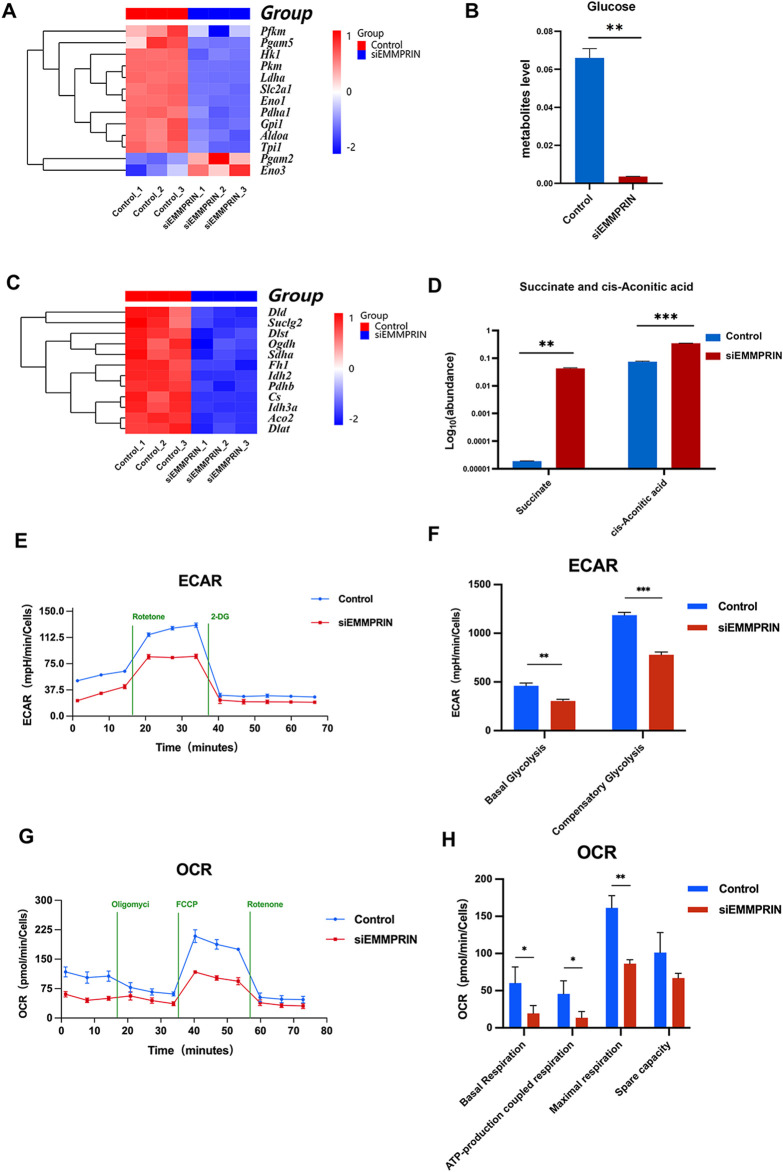
Glucose metabolism changed. **(A)** The expression levels of genes associated with glycolysis were altered. **(B)** Glucose levels were significantly downregulated in metabolites. **(C)** Genes associated with the TCA cycle showed a decreasing trend. **(D)** Succinate and cis-aconitic acid were upregulated in the TCA metabolites. **(E–H)** OCR and ECAR were decreased in the siE group (n = 6 for each group).

Functional terms related to glycolysis and the tricarboxylic acid cycle (TCA) were enriched in both the transcriptome and metabolome results. The expressions of glycolytic related genes Hk1, Gpi1, Slc2a1, Pkm, Ldha and Pfkm showed a downward trend in siE group, while the expressions of Pgam2 and Eno3 showed an upward trend (Figure 6A). Besides, the glucose levels were significantly downregulated in metabolites, which may indicate a decline in glucose demand and utilization levels (Figure 6B). However, no significant difference was found in the content of intermediates of glycolysis. Genes associated with the TCA cycle showed a decreasing trend, including Aco2, Idh2, Idh3a, Dld, Dlst, Suclg2, Dlat, etc (Figure 6C). Furthermore, succinate and cis-aconitic acid were significantly upregulated among the TCA metabolites (Figure 6D). In order to further determine the glucose utilization of BMDMs in the siE group, the Seahorse Extracellular Flow Analyzer was used to detect OCR and ECAR. Our results showed that the levels of basal glycolysis (the glycolysis rate at steady state), compensatory glycolysis (the glycolysis rate after blocking OXPHOS), basal respiration (the respiration rate at steady state), ATP-production coupled respiration, maximal respiration (the respiration rate under stress state), and spare respiration capacity were all significantly decreased in the siE group, indicating that siEMMPRIN reduced the levels of glycolysis and oxidative phosphorylation (OXPHOS) in BMDMs (Figures 6E–H).

**FIGURE 7:**
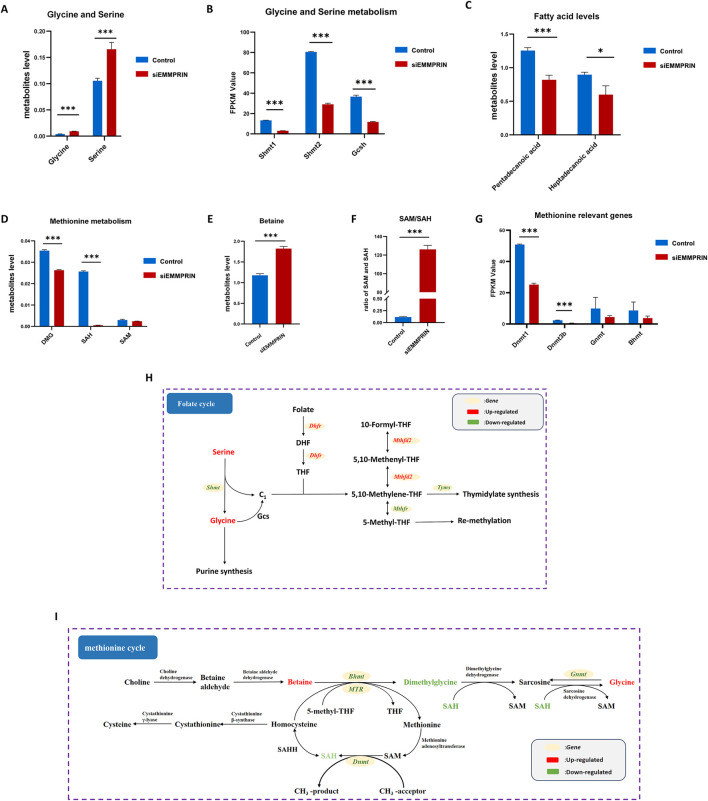
Modifications in lipid metabolism and inhibition of BMDM cell proliferation. **(A,B)** Pparg, Rxra, Lxra, Abca1, and Abcg1 were upregulated. **(C)** Fatty acid levels were downregulated. **(D)** Genes related to fatty acid synthesis exhibited a trend towards downregulation. **(E)** Oil red O staining showed decreased intracellular lipid levels (n = 6 for each group). **(F)** Genes related to the cell cycle were significantly downregulated. **(G)** Flow cytometry confirmed cell cycle arrest (n = 6 for each group). **(H)** Genes related to DNA replication were consistently downregulated. **(I)** CCK-8 test showed the inhibition of cell proliferation of BMDMs (n = 6 for each group).

The transcriptome results showed enrichment of the PPAR signaling pathway and ABC transporters in the siE group, and upregulation of gene expression of Pparg (encoding PPARγ), Rxra, Nr1h3 (encoding LXRα), Abca1, and Abcg1 (Figures 7A, B). The PPARγ-LXRα-ABCA1/G1 pathway plays a key facilitator role in regulating cholesterol efflux, thereby suggesting that cholesterol efflux may be increased in the siE group.

Moreover, fatty acid levels were significantly downregulated, and genes related to fatty acid synthesis (FAS), such as Fasn, Acaca, Acacb, Acsl1, and Mecr, exhibited a trend towards downregulation (Figures 7C, D). However, no significant changes in fatty acid oxidation-related genes were observed. These findings suggested reduced fatty acid storage preference. Consistently, oil red O staining showed a significant decrease in intracellular lipid levels, further suggesting that EMMPRIN inhibition resulted in a significant reduction in intracellular lipids in BMDMs (Figure 7E).

### 3.5 Inhibition of cell proliferation of BMDMs from AS mouse induced by siEMMPRIN intervention

Our results showed that genes related to cell cycle were significantly altered in the siE group, including 19 were upregulated genes and 102 were downregulated genes (Figure 2B). These results may suggest overall cell cycle inhibition. Among them, cyclin dependent kinases (CDKs) and cylin (encoded by Ccn) play an essential role in regulating the cell cycle, with cell division cycle 25 (CDC25) being the upstream regulator. CDK1, CDK2, CDK4, CDK6, Cyclin B1, Cyclin D, Cyclin E, CDC25A, CDC25B, and CDC25C were downregulated in the siE group, indicating BMDMs cell cycle may arrest (Figure 7F). Flow cytometry was used to detect the cell cycle, which confirmed cell cycle arrest at the G0/G1 phases in BMDMs of the siE group (Figure 7G). DNA replication plays a central role in cell proliferation, and upregulation of related genes is necessary for cell proliferation into the S phase. In the siE group, genes related to DNA replication, such as Pol, Mcm, Orc, Rpa, Rfc, Pif1, Lig1, and Fen1 were consistently downregulated, suggesting that DNA replication was widely inhibited in BMDMs (Figure 7H). The CCK-8 results indicated that the proliferation of BMDMs was significantly reduced in the siE group (Figure 7I).

Collectively, this demonstrates that siEMMPRIN caused significant inhibition of cell cycle progression and cell proliferation in BMDMs from AS mice.

## 4 Discussion

To elucidate the role of EMMPRIN and its associated pathways in the development of AS, integrated metabolomic and transcriptomic analyses were performed to investigate the cellular and molecular responses of BMDMs in AS mice after exposure to EMMPRIN siRNA *in vivo*. The metabolomic analysis revealed enriched nucleotide, amino acid metabolism (mainly methionine cycle and glycine metabolism), glucose metabolism, and lipid metabolism. In addition, transcriptomic data demonstrated that the cell cycle, DNA replication, the generation and utilization of 1C units and *de novo* nucleotide synthesis of BMDMs were significantly influenced. The effect of EMMPRIN on cell cycle progression and proliferation was verified in further experiments. More importantly, the integrated metabolomic and transcriptomic analysis suggested that EMMPRIN primarily affected nucleotide and 1C metabolism. Moreover, the Seahorse assay and oil red O staining further confirmed the effect of EMMPRIN on glucose metabolism and lipid metabolism of BMDMs. Our study contributes to a better understanding of the role of EMMPRIN in AS through multi-omics analysis.

Evidence indicated that monocytes and macrophages play essential roles in atherosclerotic disease, promoting foam cell formation and inflammatory response. In the development of AS, blood monocytes enter the artery wall and differentiate into macrophages (Wang et al., 2022). Circulating monocytosis is an independent risk factor in the development of AS (Getz et al., 2019). Moreover, Rahman et al. (2017) showed that the hypercholesterolaemic environment and the disruption of cholesterol efflux promote the proliferation of hematopoietic progenitor cells (HSPCs) and monocytosis. These contribute to the inflamed atherosclerotic lesions with neutrophils and macrophages. Reducing white blood cell count and inflammatory phenotype, particularly in terms of monocytes and macrophages, may reduce cardiovascular disease (CVD) risk and promote AS regression (Barrett et al., 2019). Therefore, the proliferation capacity of monocytes *in vivo* may be an important influencing factor in AS. Our previous studies have indicated that EMMPRIN can regulate the proliferation and the alteration of differentiation outcomes in THP-1 and RAW264.7 macrophages with oxidized low density lipoprotein (oxLDL) treatment (Liang et al., 2018). Other studies have also reported that EMMPRIN can affect the proliferation of tumor cells (Zhu et al., 2015). However, the effect and mechanism of EMMPRIN on BMDMs proliferation remains poorly understood.

In our study, the transcriptomic results suggested significant alterations in genes associated with the cell cycle within the siE group. CDK1, CDK2, CDK4, CDK6, Cyclin B1, Cyclin D, Cyclin E, CDC25A, CDC25B, and CDC25C were markedly downregulated. Cyclins and CDKs play essential roles in regulating the cell division cycle (Benot-Dominguez et al., 2022). During the G1 phase, cyclin D binds with Cdk4/CD6, while cyclin E forms a complex with CDK2 (Lim and Kaldis, 2013). The CDK4/6-cyclin D complex is responsible for phosphorylating the retinoblastoma protein (Rb). This phosphorylation is commonly believed to weaken the interaction between Rb and the transcription factor heterodimer E2F/DP (collectively termed E2F), leading to the activation of CDK2-cyclin E and the transition into the S phase (van den Heuvel and Dyson, 2008). The cyclin B1/CDK1 complex is a key regulatory factor for the G2/M transition, capable of phosphorylating various proteins before the G2/M transition, thereby initiating mitotic events of the M phase (Wang et al., 2014). CDC25 phosphatases are crucial factors in eukaryotic cell cycle control (Abdelwahab et al., 2022). They are responsible for dephosphorylating CDKs at specific stages of the cell cycle, serving as upstream regulators of CDKs and the cyclin family. CDC25A, CDC25B, and CDC25C are the three main subtypes of the CDC25 phosphatase family. CDC25A controls the G1/S transition by inducing the dephosphorylation of CDK2-cyclin E and CDK4/6-cyclin D (Shen and Huang, 2012), while CDC25B and CDC25C activate the cyclin B1/CDK1 complex to promote the G2/M transition (Liu et al., 2020). In siEMMPRIN-intervened AS mouse BMDMs, the transcription of CKDs and cyclins is inhibited and the expression levels of CDC25 are suppressed, suggesting a possible decrease in the G1/S and G2/M transitions in BMDMs. This hypothesis was confirmed by further flow cytometry analysis. Thus, siEMMPRIN may inhibit the cell cycle progression of mouse BMDMs through the G1/S and G2/M checkpoints. Additionally, genes associated with DNA replication, including DNA polymerases (Pol), minichromosome maintenance (MCM) proteins, origin recognition complex (ORC), replication protein A (RPA), replication factor C (RFC) family, as well as many others such as pif1, DNA ligase I (LIG1), and flap endonuclease I (FEN1), were significantly downregulated in the siE group. This indicates that the processes of DNA unwinding, synthesis, and replication are adversely affected in BMDMs with suppressed EMMPRIN expression. DNA replication plays a central role in cell proliferation, and CCK8 assays confirmed that siEMMPRIN decreased the proliferative capacity of BMDMs in AS mice. These findings may be attributed to a combined effect of cell cycle arrest and reduced DNA replication. Our previous studies have indicated that adenoviral intervention inhibiting EMMPRIN expression in AS mouse alleviated their plaque burden, which was correlated with reduced macrophage and lipid deposition within plaques and attenuated inflammation. Since BMDMs are a significant source of macrophages within plaques, the proliferative inhibition of AS mouse BMDMs induced by siEMMPRIN may be a mechanism underlying plaque alleviation.

Integrated omics analysis suggests that suppression of EMMPRIN gene expression leads to significant downregulation of genes related to *de novo* synthesis pathways of purine and pyrimidine nucleotides in BMDMs. These include purine-related genes Prps1, Prps2, Ppat, Gart, Pfas, Paics, Adsl, and Atic, as well as pyrimidine-related genes Dhodh, Umps, Ctps, Ctps2, and Tyms. Meanwhile, salvage synthesis pathway-related genes show no significant changes. Both purines and pyrimidines are important components of nucleotides, which are the basic units for DNA and RNA synthesis (Minchin and Lodge, 2019). Nucleotide metabolism is closely associated with the cell cycle (Ali and Ben-Sahra, 2023). During cell proliferation, there is a need for increased nucleotide synthesis to accomplish DNA replication and RNA production, and genes associated with nucleotide synthesis are upregulated in late G1 phase (Lane and Fan, 2015). Nucleotide synthesis occurs through two different pathways: *de novo* synthesis and salvage pathways. Fast-paced cell proliferation is typically more closely linked with *de novo* synthesis pathways (Li et al., 2023). The significant downregulation of genes related to *de novo* synthesis pathways suggests that EMMPRIN inhibition may severely affect *de novo* nucleotide synthesis in BMDMs. Furthermore, the proto-oncogene MYC is a major regulator of nucleotide metabolism as it directly controls the expression of pyrimidine and purine synthesis genes (Liu YC. et al., 2008). The significant decrease in MYC expression in the siE group may reduce the expression of nucleotide synthesis genes, consistent with the inhibition of *de novo* synthesis pathways. PPP provides ribose for nucleic acid biosynthesis. PPP is also associated with the inflammatory function of macrophages within AS plaques (Bories and Leitinger, 2017). In the siE group, key enzymes of the PPP, including glucose-6-phosphate dehydrogenase (G6PD) and hexose-6-phosphate dehydrogenase (H6PD), were significantly downregulated, indicating inhibition of the PPP and impairment of its role in providing ribose as a substrate. Meanwhile, metabolomics also showed a significant decrease in the level of ribose. Therefore, siEMMPRIN intervention may inhibit nucleotide synthesis in BMDMs, suggesting that EMMPRIN may be an indispensable molecule in the nucleotide synthesis process of BMDMs. On the other hand, transcriptomic analysis suggests that genes associated with purine degradation, including Pnp2, Lacc1, Gda, and Xdh, were significantly upregulated in the siE group, while metabolomic analysis indicates an increase in purine degradation metabolites such as xanthene and urate. However, no significant changes were observed in genes and metabolites related to pyrimidine nucleotide degradation pathways. These suggests a correlation between EMMPRIN and purine nucleotide degradation. When EMMPRIN expression is suppressed, there is an increase in degradation, which could potentially reduce available nucleotides and negatively affect cell proliferation.

Curiously, despite the decrease in nucleotide synthesis and increase in degradation observed in AS mouse BMDMs upon EMMPRIN inhibition, metabolomics reveal a significant accumulation of nucleosides, nucleotides, and nucleobases. GMP, AMP, UMP, IMP, dTMP, dAMP, and dGMP are notably upregulated among nucleotides, while thymidine, uridine, deoxycytidine, deoxyuridine, inosine, and adenosine show increased levels among nucleosides. Thymine, cytosine, and guanine also show upregulation in nucleobases. In cases where de novo nucleotide synthesis is inhibited, this accumulation may indicate reduced utilization of nucleosides and nucleotides. The activities of several enzymes involved in nucleotide biosynthesis are regulated by allosteric interactions and inhibited by feedback of related pathway products. For instance, the activities of enzymes phosphoribosyl pyrophosphate amidotransferase (PPAT) and phosphoribosyl pyrophosphate synthetase (PRPS) are inhibited by AMP, GMP (Fridman et al., 2013), while cytidine 5′-triphosphate synthaseII (CPSII) is inhibited by UMP (Sigoillot et al., 2003). Therefore, the accumulation of nucleotides may further inhibit key enzymes involved in *de novo* synthesis of purines and pyrimidines through negative feedback mechanisms. Increased degradation of purine nucleotides but not pyrimidine nucleotides is observed upon EMMPRIN suppression. There is no significant difference in the accumulation of purine and pyrimidine nucleotides. This suggests that the inhibitory effect of siEMMPRIN on *de novo* nucleotide synthesis in BMDMs may far outweigh its effect on increasing nucleotide degradation. Additionally, while salvage synthesis pathway-related gene expression remains unchanged in the siE group, significant decreases are observed in RNR, AMK, GMK and NDPK. During the S phase, cells utilize RNR to convert NTP to dNTP to facilitate DNA synthesis (Buj and Aird, 2018). Thus, the decreased expression of RNR in the siE group may affect this process, influencing cell proliferation into the S phase. Moreover, nucleosides or nucleobases are converted into homologous NMP through single phosphorylation or phosphoribosyl transferase reactions. NMPK promotes the conversion of NMP to NDP, while NDPK promotes the conversion of NDP to NTP (Ray and Hostetler, 2011). Therefore, decreased expression levels of NMPK and NDPK in the siE group may lead to reduced utilization of NMP, as well as decreased NDP and NTP production. In brief, inhibition of EMMPRIN expression may reduce the conversion of NMP to NTP and dNTP, leading to decreased nucleotide utilization, which may further exacerbate nucleotide accumulation. Additionally, John et al. (2023) demonstrated that in the late stage of inflammation stimulation, macrophages accumulate pentose phosphates, nucleotides, nucleosides, nitrogencontaining bases, ribose, which correlates with the transition of macrophages to an anti-inflammatory phenotype over time. This is characterized by an increase in anti-inflammatory cytokines and a decrease in pro-inflammatory cytokines, suggesting that nucleotide metabolism restructuring may also be associated with macrophage functional phenotype transition. Recently, Lv et al. (2024) showed that myeloid-restricted EMMPRIN overexpression promotes macrophage transition to an inflammatory phenotype in ApoE−/− mice. Our previous studies also indicated that EMMPRIN overexpression induces RAW264.7 cells towards inflammatory phenotype, while EMMPRIN suppression promotes their transition toward anti-inflammatory phenotype. In conclusion, the nucleotide metabolism restructuring induced by siEMMPRIN in this study may also impact the functional phenotype of BMDMs, which requires further experimental validation.

Multi-omics analysis also highlighted significant changes in 1C metabolism in the siE group. KEGG enrichment revealed significant alterations in three pathways: glycine/serine/threonine metabolism; cysteine/methionine metabolism; and folate metabolism. Transcriptomic results showed decreased genes expression levels of enzymes involved in glycine and serine degradation, including SHMT1, SHMT2, and GCS. Genes related to folate metabolism enzymes thymidylate synthase (TYMS), methylenetetrahydrofolate dehydrogenase (MTHFD2), and dihydrofolate reductase (DHFR) showed downregulation, while methylenetetrahydrofolate reductase (MTHFR) increased. Genes related to enzymes involved in the methionine cycle were affected, including DNA methyltransferases (DNMTs), betaine-homocysteine methyltransferase (BHMT), and glycine N-methyltransferase (GNMT). Metabolomic data showed increased levels of serine and glycine. Betaine levels related to the methionine cycle increased, while levels of DMG and SAH decreased, accompanied by a declining trend in SAM. These changes are closely related to 1C metabolism. 1C metabolism, mediated by folate, supports various physiological processes through the generation and transfer of 1C units (Lan et al., 2018). This includes interconversion of glycine and serine, nucleotide biosynthesis (including purine and thymidine synthesis), and remethylation of homocysteine (HCY). Serine and glycine are the main sources of 1C units (Ducker and Rabinowitz, 2017). Serine is converted to glycine via SHMT, generating 1C units. Glycine can directly participate in *de novo* synthesis of purine nucleotides or generate 1C units through GCS (Reina-Campos et al., 2020). Therefore, the decrease in SHMT and GCSH levels, along with the increase in serine and glycine levels, suggests reduced utilization of glycine and serine in one-carbon metabolism within BMDMs after EMMPRIN inhibition, which may decrease the production of 1C units.

1C units are typically carried and metabolized by tetrahydrofolate (THF) and do not exist freely in the body (Newman and Maddocks, 2017). THF is sequentially formed by folate through DHFR and is the biologically active form of folate in the body. Once bound to THF, 1C units can exist in three main carbon oxidation states, such as 5,10-methylene-THF, 5-methyl-THF, and 10-formyl-THF. Each of these states plays specific biosynthetic roles through different covalent bonds to these nitrogen atoms. 5,10-Methylene-THF, the most basic form of the 1C unit, acts as a methyl donor and is involved in the conversion of dUMP to dTMP by TYMS, affecting nucleoside synthesis (Ducker and Rabinowitz, 2017). Meanwhile, 10-formyl-THF, which is formed by the oxidation of 5,10-methylene-THF by MTHFD2, is essential for the *de novo* synthesis pathway of purines (Ducker and Rabinowitz, 2017). Therefore, 1C units participate in the synthesis of thymidine and purines, among others, which have significant effects on cell proliferation. When genes related to Tyms, Mthfd2, and Dhfr are downregulated, the production and utilization of 1C units are affected, suggesting that EMMPRIN expression inhibition may have an inhibitory effect on the generation and transformation of 1C units in BMDMs of AS model mice.

5-Methyl-THF, catalyzed by MTHFR from 5,10-methylene-THF, serves as the most reduced form of folate 1C unit. Its significant role is to participate in the remethylation of HCY to produce methionine (Kim et al., 2023). Methionine serves as a substrate for SAM synthase. SAM is the most important methyl donor for methyltransferases (such as DNMT) to maintain methylation patterns in macromolecules (such as DNA and histones) (Murín et al., 2017). Methylation reactions are closely related to the cell cycle and proliferation. Reduced methylation is associated with low SAM levels and elevated levels of the methylation reaction product SAH, and the SAM/SAH ratio is used to measure methylation levels (Hoffmann and Schulz, 2005). Yan et al. (2019) suggested that SAM affects the pathways of the cell cycle and inhibits the proliferation of liver cells. Increased levels of SAH have been closely associated with the formation of AS-related plaques in animal models and cell experiments (Liu C. et al., 2008). Zhang et al. (2016) proposed that the severity of atherosclerotic lesions in ApoE−/− mice is related to the SAM/SAH ratio and suggested using the SAM/SAH ratio as a biomarker to provide sensitive indicators for the clinical diagnosis of AS. Recent evidence also suggests that SAM can influence methylation levels in macrophages and exert anti-inflammatory effects by reducing the expression of the pro-inflammatory cytokine tumor necrosis factor alpha (TNFα) and increasing the expression of the anti-inflammatory cytokine interleukin 10 (IL-10) (Pfalzer et al., 2014). In our study, SAH levels decreased and the SAM/SAH ratio increased in the siE group, which may have an important impact on BMDMs methylation and AS development. Additionally, mammalian cells can convert betaine to DMG through BHMT to achieve HYC remethylation in a folic acid-independent manner (Ducker and Rabinowitz, 2017). Betaine, a chemical compound found in the pathway from choline to glycine, can lower SAH levels by promoting HYC remethylation (Radziejewska et al., 2020). Dai et al. (2022) has shown that supplementing betaine can reduce SAH levels, thus mitigating the effects of high SAH on AS in ApoE−/− mice. DMG, a derivative of glycine, plays a key role in methylation and transmethylation processes within the body. McGregor et al. (2001) indicate that plasma DMG levels are positively correlated with HCY levels, which may influence HCY remethylation. Furthermore, elevated plasma levels of DMG have been reported to correlate positively with the risk of atherosclerotic cardiovascular disease (Zaric et al., 2020). The increase in betaine levels and the decrease in SAH and DMG levels in the siE group suggest that the suppression of EMMPRIN expression in AS model mice may reduce plaque formation, potentially linked to the elevated levels of betaine and the reduced levels of DMG in BMDMs.

In addition to one-carbon metabolism, energy metabolism, such as glycolysis and lipid metabolism, is closely related to cell function and status. Multi-omics results enriched terms related to glycolysis and the TCA cycle in the siE group. Glucose metabolism is fundamental to the physiological balance of organisms, which can provide energy to cells and maintain various physiological processes. In the pathological setting of AS, reservoir and circulating hematopoietic myeloid populations are stimulated to undergo metabolic reprogramming. This process is characterized by enhanced glycolysis, elevated OXPHOS rates, and notably, an inflammatory phenotype (Koelwyn et al., 2018). Sarrazy et al. (2016) suggest that increased glucose transporter 1 (Glut1) -dependent glucose uptake is associated with increased bone marrow hematopoiesis and monocytic generation, subsequently leading to macrophage-dependent atherosclerotic plaque formation. Su et al. (2016) found that EMMPRIN interacts with Glut1 in A375 cells, reducing glucose uptake and inhibiting tumor cell proliferation. Our multi-omics results showd a declining trend in key genes involved in glycolysis processes such as Slc2a1 (encoding Glut1), Hk1, Gpi1, Pkm, Ldha, and Pfkm. The significant decrease in glucose levels in metabolites suggests a possible reduction in glucose uptake and storage. Genes related to key processes in the TCA cycle, including Aco2, Idh2, Idh3a, Dld, Dlst, Suclg2, and Dlat, showed a downward trend. The TCA metabolite cis-aconitic acid significantly increased, suggesting possible TCA inhibition. These results indicate that the absence of EMMPRIN affects the glucose metabolism of BMDMs in AS model mice, potentially leading to decreased cellular glucose uptake, glycolysis, and TCA metabolism. Our Seahorse assay results further confirmed significant inhibition of glycolysis and OXPHOS rates in BMDMs of the siE group, indicating reduced energy supply to BMDMs, which could have significant implications for cell proliferation status and functional phenotype. This may be one of the mechanisms by which siEMMRPIN alleviates plaque burden in AS model mice. However, the upward trend of certain enzymes in this process, such as Pgam2 and Eno3 genes, and the increase in succinic acid levels in metabolites, are not yet fully understood and may be attributed to small sample sizes and individual variations.

The lipid metabolism of macrophages is closely associated with the formation and progression of AS plaques (Sukhorukov et al., 2020). Fernandez-Ruiz et al. (2016) found that when exposed to high levels of cholesterol in the circulation, peripheral blood monocytes accumulate lipids and may become foam cells. These foam-like monocytes adhere to and migrate into AS lesions, promoting the accumulation of lipids within atherosclerotic lesions. Our transcriptomic results indicate enrichment of the PPAR signaling pathway and ABC transporters in the siE group, with upregulation of genes such as PPARγ, RXRa, LXRα, ABCA1, and ABCG1. Lv et al. (2021) found that the deletion of the EMMPRIN gene in macrophages may inhibit cholesterol uptake via the scavenger receptor CD36, thereby protecting against foam cell formation. However, this study highlighted that the role of EMMPRIN may not be directly associated with cholesterol efflux and the ABC family. Nevertheless, our results show upregulation of ABCA1 and ABCG1 expression in the siE group. ABCA1 and ABCG1 are located in the cell membrane, mediating cholesterol efflux to high-density lipoprotein and lipid-free apolipoprotein (AI), playing a crucial role in inhibiting foam cell formation. Oil Red O staining confirmed that siEMMPRIN can reduce lipid accumulation in BMDMs. Therefore, we speculate that siEMMPRIN may affect cholesterol efflux in BMDMs, but whether this occurs through ABCA1 and ABCG1 remains to be confirmed. LXRα, RXRα, and PPARγ are common transcription factors that upregulate the expression of ABC transporters under conditions of excessive cholesterol accumulation (Sukhorukov et al., 2020). Intervention with siEMMPRIN led to the upregulation of LXRα, RXRα, and PPARγ. This could further boost the expression of ABCA1 and ABCG1, enhancing cholesterol efflux in BMDMs of AS model mice. The result is reduced intracellular lipid deposition, decreased foam cell formation, and potentially inhibited cell proliferation. Additionally, our results also indicate changes in fatty acid metabolism in the siE group: decreased levels of fatty acids, inhibition of enzymes involved in FAS such as Fasn, Acaca, Acacb, Acsl1, and Mecr, but no significant changes in genes related to fatty acid degradation. Fatty acids are the main components of various lipids and can provide acetyl-CoA for OXPHOS through β-oxidation metabolism (Guo et al., 2022). Lipid synthesis is also essential for cell proliferation and growth to produce new organelles and cell membranes (Carracedo et al., 2013). Recent studies have shown that lipid synthesis is associated with macrophage function, with enhanced lipid synthesis being related to increased macrophage phagocytosis and inflammatory function (Yan and Horng, 2020). Therefore, in the siE group, the downregulation of genes related to FAS and the decrease in fatty acid levels could impact the cellular energy supply. This change may significantly affect cell proliferation and the inflammatory phenotype. This may also be one of the mechanisms by which siEMMPRIN improves plaque formation in AS model mice. This hypothesis warrants further experimental validation.

In summary, we intervened AS animal model (ApoE−/− mice fed a high-fat diet) with EMMPRIN siRNA and studied the metabolic and transcriptional changes in their BMDMs. The integrated metabolomic and transcriptomic analysis suggested that nucleotide and 1C metabolism may be the major metabolic pathways affected by EMMPIN in AS mouse BMDMs and we suggest that SAH and SAM may be important molecules mediating the effect of EMMPRIN on BMDMs status and AS development. These findings provide new insights for further elucidating the promoting role of EMMPRIN in the development of AS and its underlying mechanisms.

## Data Availability

The metabolomics dataset presented in the study is deposited in the National Genomics Data Center - OMIX (NGDC-OMIX), accession number OMIX009237 (https://ngdc.cncb.ac.cn/omix/release/OMIX009237). The transcriptomics dataset presented in the study is deposited in the NCBI Sequence Read Archive (SRA), accession number PRJNA1223352 (https://www.ncbi.nlm.nih.gov/sra/PRJNA1223352).
